# (*E*)-1-(2,4-Dimethyl­quinolin-3-yl)-3-(4-methyl­phen­yl)prop-2-en-1-one

**DOI:** 10.1107/S1600536812015498

**Published:** 2012-04-21

**Authors:** R. Prasath, P. Bhavana, Ray J. Butcher

**Affiliations:** a Department of Chemistry, Birla Institute of Technology and Science (BITS), Pilani – K. K. Birla Goa, Campus, Zuarinagar, Goa 403 726, India; b Department of Chemistry, Howard University, 525 College Street, NW, Washington, DC 2059, USA

## Abstract

In the title compound, C_21_H_19_NO, there are two mol­ecules in the asymmetric unit (*Z*′ = 2). There are π–π inter­actions between these two mol­ecules [centroid–centroid distance = 3.678 (2) Å], as well as a weak C—H⋯O inter­action. The conformation adopted by the two mol­ecules is such that the quinoline mean plane and the benzene ring are almost perpendicular [89.04 (5) and 76.89 (4)°]. In each mol­ecule, the methyl group of the tolyl ring is disordered over two conformations, with occupancy ratios of 0.56 (3):0.44 (3) and 0.65 (3):0.35 (3).

## Related literature

For background details and biological applications of quinolines, see: Muscia *et al.* (2006 ▶); Kalluraya & Sreenivasa (1998 ▶); Campbell *et al.* (1998 ▶); Dimmock *et al.* (1999 ▶). For the anti­plasmodial, anti­microbial, anti­malarial and anti­cancer activity of quinoline chalcone analogues, see: Xiang *et al.* (2006 ▶). For a related structure, see: Prasath *et al.* (2011 ▶).
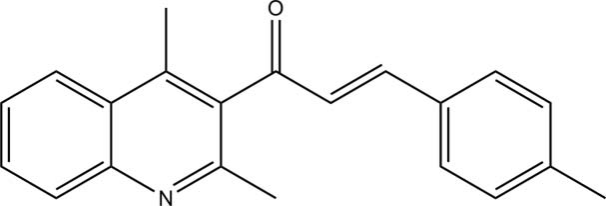


## Experimental

### 

#### Crystal data

C_21_H_19_NO
*M* *_r_* = 301.37
Triclinic, 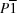

*a* = 11.4915 (4) Å
*b* = 12.0673 (5) Å
*c* = 13.0695 (6) Å
α = 111.864 (4)°
β = 92.141 (3)°
γ = 93.705 (3)°
*V* = 1674.79 (12) Å^3^
*Z* = 4
Cu *K*α radiation
μ = 0.57 mm^−1^
*T* = 295 K
0.48 × 0.23 × 0.17 mm

#### Data collection

Agilent Xcalibur Ruby Gemini diffractometer
Absorption correction: multi-scan (*CrysAlis PRO*; Agilent, 2012 ▶) *T*_min_ = 0.800, *T*_max_ = 1.000
11652 measured reflections
6514 independent reflections
5419 reflections with *I* > 2σ(*I*)
*R*_int_ = 0.022

#### Refinement

*R*[*F* ^2^ > 2σ(*F* ^2^)] = 0.048
*wR*(*F* ^2^) = 0.143
*S* = 1.04
6514 reflections
423 parameters
H-atom parameters constrained
Δρ_max_ = 0.21 e Å^−3^
Δρ_min_ = −0.20 e Å^−3^

### 

Data collection: *CrysAlis PRO* (Agilent, 2012 ▶); cell refinement: *CrysAlis PRO*; data reduction: *CrysAlis PRO*; program(s) used to solve structure: *SHELXS97* (Sheldrick, 2008 ▶); program(s) used to refine structure: *SHELXL97* (Sheldrick, 2008 ▶); molecular graphics: *SHELXTL* (Sheldrick, 2008 ▶); software used to prepare material for publication: *SHELXTL*.

## Supplementary Material

Crystal structure: contains datablock(s) I, global. DOI: 10.1107/S1600536812015498/hg5205sup1.cif

Structure factors: contains datablock(s) I. DOI: 10.1107/S1600536812015498/hg5205Isup2.hkl

Supplementary material file. DOI: 10.1107/S1600536812015498/hg5205Isup3.cml

Additional supplementary materials:  crystallographic information; 3D view; checkCIF report

## Figures and Tables

**Table 1:** Hydrogen-bond geometry (Å, °)

*D*—H⋯*A*	*D*—H	H⋯*A*	*D*⋯*A*	*D*—H⋯*A*
C16*B*—H16*B*⋯O1*A*^i^	0.93	2.63	3.4465 (19)	148

Symmetry code: (i) 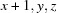.

**Fig. 1.**
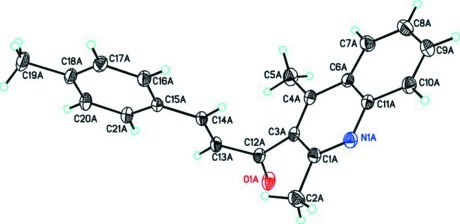
The molecular structure of C21H19NO showing the atom numbering scheme and 30% probability displacement ellipsoids (only the major component for the disordered CH3 substituent shown).

**Fig. 2.**
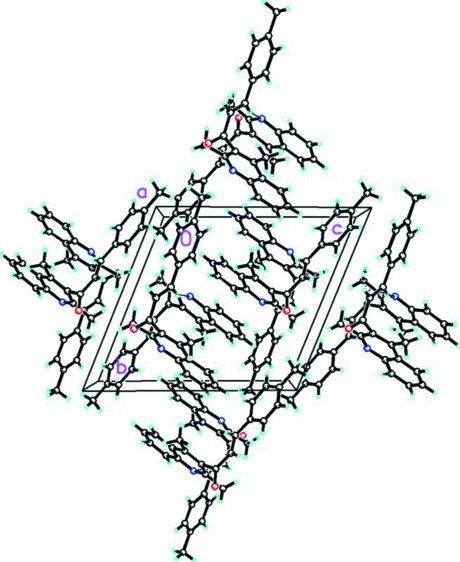
The molecular packing for C21H19NO viewed down the a axis. C—H···O interactions are shown by dashed lines (only the major component for the disordered CH3 substituent shown).

## supplementary crystallographic information

### Comment

The quinoline derivatives are very important compounds because of their wide occurrence in natural products and biologically active compounds [Muscia *et al*., (2006); Kalluraya & Sreenivasa (1998); Campbell *et al.*, (1998); Dimmock *et al.* (1999)]. Quinoline chalcone analogues have also much attention due to their bioactivity such as antiplasmodial, antimicrobial, antimalarial and anticancer activities (Xiang *et al.*, 2006).

In the title compound, C_21_H_19_NO, there are two molecules in the asymmetric unit (*Z*' = 2). There are π–π interactions between these two molecules [cg2···cg5, 3.678 (2) Å; 1 - *x*, 1 - *y*, 1 - *z*] as well as a weak C—H···O interaction. The conformation adopted by the two molecules is such that quinoline and phenyl rings are almost perpendicular [89.04 (5)° and 76.89 (4)°]. In each molecule, the methyl group attached to the phenyl ring is disordered over two conformations with occupancies of 0.56 (3)/0.44 (3) and 0.65 (3)/0.35 (3). A structure of a related quinoline chalcone analogue has recently been determined [Prasath *et al.*, (2011)].

### Experimental

A mixture of 3-acetyl-2,4-dimethylquinoline (1.0 g, 0.005 *M*), 4-methylbenzaldehyde (700 mg, 0.005 *M*) and 0.5 g of KOH in 50 ml distilled ethanol was stirred for 12 h at room temperature. The resulting mixture was neutralized with diluted acetic acid. The resultant solid was filtered, dried and purified by column chromatography using 1:3 mixture of ethyl acetate and hexane. Re-crystallization was by slow evaporation of acetone solution of (I) which yielded white colour needle type crystals. *M*.pt. 413–415 K. Yield: 86%

### Refinement

H atoms were placed in geometrically idealized positions and constrained to ride on their parent atoms with C—H distances of 0.93 and 0.96 Å *U*_iso_(H) = 1.2*U*_eq_(C) [*U*_iso_(H) = 1.5*U*_eq_(C) for CH_3_].

### Crystal data

**Table:**

C_21_H_19_NO	*Z* = 4
*M**_r_* = 301.37	*F*(000) = 640
Triclinic, *P*1	*D*_x_ = 1.195 Mg m^−^^3^
Hall symbol: -P 1	Cu *K*α radiation, λ = 1.54178 Å
*a* = 11.4915 (4) Å	Cell parameters from 6502 reflections
*b* = 12.0673 (5) Å	θ = 5.1–73.4°
*c* = 13.0695 (6) Å	µ = 0.57 mm^−^^1^
α = 111.864 (4)°	*T* = 295 K
β = 92.141 (3)°	Chunk, colorless
γ = 93.705 (3)°	0.48 × 0.23 × 0.17 mm
*V* = 1674.79 (12) Å^3^	

### Data collection

**Table:**

Agilent Xcalibur Ruby Gemini diffractometer	6514 independent reflections
Radiation source: Enhance (Cu) X-ray Source	5419 reflections with *I* > 2σ(*I*)
Graphite monochromator	*R*_int_ = 0.022
Detector resolution: 10.5081 pixels mm^-1^	θ_max_ = 73.6°, θ_min_ = 5.1°
ω scans	*h* = −12→14
Absorption correction: multi-scan (*CrysAlis PRO*; Agilent, 2012)	*k* = −13→14
*T*_min_ = 0.800, *T*_max_ = 1.000	*l* = −15→16
11652 measured reflections	

### Refinement

**Table:**

Refinement on *F*^2^	Primary atom site location: structure-invariant direct methods
Least-squares matrix: full	Secondary atom site location: difference Fourier map
*R*[*F*^2^ > 2σ(*F*^2^)] = 0.048	Hydrogen site location: inferred from neighbouring sites
*wR*(*F*^2^) = 0.143	H-atom parameters constrained
*S* = 1.04	*w* = 1/[σ^2^(*F*_o_^2^) + (0.0752*P*)^2^ + 0.1557*P*] where *P* = (*F*_o_^2^ + 2*F*_c_^2^)/3
6514 reflections	(Δ/σ)_max_ < 0.001
423 parameters	Δρ_max_ = 0.21 e Å^−^^3^
0 restraints	Δρ_min_ = −0.20 e Å^−^^3^

### Special details

**Table:**

Geometry. All e.s.d.'s (except the e.s.d. in the dihedral angle between two l.s. planes) are estimated using the full covariance matrix. The cell e.s.d.'s are taken into account individually in the estimation of e.s.d.'s in distances, angles and torsion angles; correlations between e.s.d.'s in cell parameters are only used when they are defined by crystal symmetry. An approximate (isotropic) treatment of cell e.s.d.'s is used for estimating e.s.d.'s involving l.s. planes.
Refinement. Refinement of *F*^2^ against ALL reflections. The weighted *R*-factor *wR* and goodness of fit *S* are based on *F*^2^, conventional *R*-factors *R* are based on *F*, with *F* set to zero for negative *F*^2^. The threshold expression of *F*^2^ > σ(*F*^2^) is used only for calculating *R*-factors(gt) *etc*. and is not relevant to the choice of reflections for refinement. *R*-factors based on *F*^2^ are statistically about twice as large as those based on *F*, and *R*- factors based on ALL data will be even larger.

### Fractional atomic coordinates and isotropic or equivalent isotropic displacement parameters (Å^2^)

**Table:**

	*x*	*y*	*z*	*U*_iso_*/*U*_eq_	Occ. (<1)
O1A	−0.02167 (12)	0.33913 (11)	0.87572 (12)	0.0842 (4)	
N1A	0.30059 (10)	0.22155 (11)	0.72192 (11)	0.0589 (3)	
C1A	0.22871 (12)	0.29451 (12)	0.78377 (12)	0.0551 (3)	
C2A	0.26735 (18)	0.36072 (18)	0.90311 (16)	0.0839 (5)	
H2AA	0.3476	0.3486	0.9153	0.126*	
H2AB	0.2197	0.3313	0.9479	0.126*	
H2AC	0.2594	0.4447	0.9226	0.126*	
C3A	0.11720 (11)	0.31014 (11)	0.74209 (12)	0.0496 (3)	
C4A	0.08194 (12)	0.24848 (12)	0.63295 (13)	0.0546 (3)	
C5A	−0.03464 (16)	0.26314 (19)	0.58525 (18)	0.0845 (6)	
H5AA	−0.0745	0.3208	0.6417	0.127*	
H5AB	−0.0808	0.1875	0.5578	0.127*	
H5AC	−0.0230	0.2904	0.5259	0.127*	
C6A	0.15825 (12)	0.16739 (12)	0.56507 (12)	0.0543 (3)	
C7A	0.13133 (18)	0.09595 (18)	0.45206 (15)	0.0801 (5)	
H7AA	0.0606	0.1016	0.4181	0.096*	
C8A	0.2077 (2)	0.0190 (2)	0.39237 (16)	0.0922 (6)	
H8AA	0.1888	−0.0268	0.3180	0.111*	
C9A	0.31316 (19)	0.00816 (18)	0.44144 (16)	0.0827 (5)	
H9AA	0.3639	−0.0457	0.4002	0.099*	
C10A	0.34264 (15)	0.07544 (16)	0.54893 (15)	0.0706 (4)	
H10A	0.4141	0.0681	0.5806	0.085*	
C11A	0.26625 (12)	0.15692 (12)	0.61391 (12)	0.0527 (3)	
C12A	0.03454 (13)	0.38482 (13)	0.82204 (13)	0.0571 (3)	
C13A	0.02121 (13)	0.50819 (12)	0.83349 (14)	0.0599 (4)	
H13A	−0.0376	0.5479	0.8759	0.072*	
C14A	0.08865 (11)	0.56689 (11)	0.78652 (11)	0.0501 (3)	
H14A	0.1476	0.5258	0.7455	0.060*	
C15A	0.07944 (11)	0.68980 (11)	0.79282 (11)	0.0496 (3)	
C16A	0.16480 (14)	0.74252 (13)	0.74872 (14)	0.0634 (4)	
H16A	0.2267	0.6994	0.7161	0.076*	
C17A	0.15902 (16)	0.85811 (14)	0.75266 (15)	0.0687 (4)	
H17A	0.2169	0.8912	0.7222	0.082*	
C18A	0.06895 (14)	0.92528 (13)	0.80099 (14)	0.0619 (4)	
C19A	0.0640 (2)	1.05275 (15)	0.80823 (19)	0.0853 (6)	
H19A	0.1350	1.0782	0.7838	0.128*	0.44 (3)
H19B	0.0550	1.1041	0.8834	0.128*	0.44 (3)
H19C	−0.0012	1.0570	0.7621	0.128*	0.44 (3)
H191	0.0725	1.0556	0.7364	0.128*	0.56 (3)
H192	0.1261	1.1029	0.8586	0.128*	0.56 (3)
H193	−0.0098	1.0808	0.8342	0.128*	0.56 (3)
C20A	−0.01602 (14)	0.87276 (13)	0.84446 (16)	0.0674 (4)	
H20A	−0.0776	0.9164	0.8772	0.081*	
C21A	−0.01183 (13)	0.75715 (13)	0.84056 (14)	0.0603 (4)	
H21A	−0.0706	0.7241	0.8702	0.072*	
O1B	0.72299 (11)	0.56330 (12)	0.81047 (12)	0.0843 (4)	
N1B	0.36155 (10)	0.51646 (11)	0.68459 (11)	0.0589 (3)	
C1B	0.45650 (12)	0.52308 (13)	0.74621 (12)	0.0567 (3)	
C2B	0.45936 (18)	0.6086 (2)	0.86457 (16)	0.0900 (6)	
H2BA	0.3828	0.6346	0.8816	0.135*	
H2BB	0.5134	0.6767	0.8755	0.135*	
H2BC	0.4838	0.5689	0.9122	0.135*	
C3B	0.55180 (12)	0.45371 (13)	0.70469 (12)	0.0536 (3)	
C4B	0.54685 (13)	0.37715 (13)	0.59601 (13)	0.0557 (3)	
C5B	0.64755 (17)	0.30462 (18)	0.54707 (17)	0.0809 (5)	
H5BA	0.7123	0.3262	0.6012	0.121*	
H5BB	0.6705	0.3208	0.4838	0.121*	
H5BC	0.6237	0.2208	0.5251	0.121*	
C6B	0.44437 (13)	0.36860 (12)	0.52804 (12)	0.0552 (3)	
C7B	0.4270 (2)	0.29235 (17)	0.41557 (14)	0.0783 (5)	
H7BA	0.4853	0.2442	0.3816	0.094*	
C8B	0.3259 (2)	0.2886 (2)	0.35629 (16)	0.0971 (7)	
H8BA	0.3158	0.2374	0.2825	0.116*	
C9B	0.2376 (2)	0.3601 (2)	0.40442 (17)	0.0892 (6)	
H9BA	0.1692	0.3570	0.3627	0.107*	
C10B	0.25089 (15)	0.43406 (17)	0.51173 (15)	0.0703 (4)	
H10B	0.1914	0.4817	0.5434	0.084*	
C11B	0.35403 (12)	0.44003 (13)	0.57649 (12)	0.0535 (3)	
C12B	0.65846 (13)	0.47068 (15)	0.78172 (14)	0.0616 (4)	
C13B	0.68221 (13)	0.37728 (15)	0.82388 (14)	0.0621 (4)	
H13B	0.7542	0.3843	0.8618	0.075*	
C14B	0.60828 (12)	0.28281 (13)	0.81199 (12)	0.0542 (3)	
H14B	0.5370	0.2763	0.7732	0.065*	
C15B	0.62823 (12)	0.18851 (13)	0.85401 (11)	0.0516 (3)	
C16B	0.72983 (13)	0.18898 (13)	0.91628 (12)	0.0569 (3)	
H16B	0.7876	0.2519	0.9333	0.068*	
C17B	0.74554 (13)	0.09718 (14)	0.95279 (12)	0.0592 (3)	
H17B	0.8137	0.0998	0.9945	0.071*	
C18B	0.66212 (14)	0.00097 (14)	0.92884 (12)	0.0591 (3)	
C19B	0.68130 (18)	−0.09819 (16)	0.96933 (16)	0.0758 (5)	
H19D	0.7311	−0.0674	1.0362	0.114*	0.65 (3)
H19E	0.7175	−0.1607	0.9141	0.114*	0.65 (3)
H19F	0.6075	−0.1297	0.9836	0.114*	0.65 (3)
H194	0.6650	−0.0727	1.0458	0.114*	0.35 (3)
H195	0.7610	−0.1176	0.9611	0.114*	0.35 (3)
H196	0.6301	−0.1677	0.9269	0.114*	0.35 (3)
C20B	0.56134 (14)	0.00074 (15)	0.86715 (14)	0.0653 (4)	
H20B	0.5042	−0.0628	0.8496	0.078*	
C21B	0.54392 (13)	0.09288 (15)	0.83106 (13)	0.0608 (4)	
H21B	0.4748	0.0909	0.7908	0.073*	

### Atomic displacement parameters (Å^2^)

**Table:**

	*U*^11^	*U*^22^	*U*^33^	*U*^12^	*U*^13^	*U*^23^
O1A	0.0898 (8)	0.0635 (7)	0.1166 (10)	0.0178 (6)	0.0478 (8)	0.0481 (7)
N1A	0.0482 (6)	0.0585 (7)	0.0682 (8)	0.0098 (5)	−0.0016 (5)	0.0214 (6)
C1A	0.0543 (7)	0.0464 (7)	0.0622 (8)	0.0029 (6)	0.0003 (6)	0.0181 (6)
C2A	0.0833 (12)	0.0790 (12)	0.0706 (11)	0.0091 (9)	−0.0132 (9)	0.0078 (9)
C3A	0.0494 (7)	0.0361 (6)	0.0656 (8)	0.0043 (5)	0.0062 (6)	0.0215 (6)
C4A	0.0485 (7)	0.0475 (7)	0.0695 (9)	0.0080 (5)	0.0000 (6)	0.0237 (6)
C5A	0.0623 (9)	0.0856 (12)	0.0977 (14)	0.0237 (9)	−0.0118 (9)	0.0243 (10)
C6A	0.0566 (7)	0.0484 (7)	0.0594 (8)	0.0092 (6)	0.0007 (6)	0.0215 (6)
C7A	0.0861 (12)	0.0806 (12)	0.0641 (10)	0.0207 (9)	−0.0089 (8)	0.0152 (9)
C8A	0.1153 (16)	0.0880 (13)	0.0602 (10)	0.0281 (12)	0.0048 (10)	0.0096 (9)
C9A	0.0950 (13)	0.0779 (11)	0.0755 (11)	0.0350 (10)	0.0266 (10)	0.0227 (9)
C10A	0.0642 (9)	0.0734 (10)	0.0794 (11)	0.0261 (8)	0.0158 (8)	0.0302 (9)
C11A	0.0509 (7)	0.0482 (7)	0.0617 (8)	0.0095 (5)	0.0065 (6)	0.0227 (6)
C12A	0.0561 (7)	0.0463 (7)	0.0730 (9)	0.0067 (6)	0.0140 (7)	0.0257 (7)
C13A	0.0624 (8)	0.0446 (7)	0.0752 (9)	0.0161 (6)	0.0235 (7)	0.0220 (7)
C14A	0.0508 (7)	0.0425 (6)	0.0559 (7)	0.0105 (5)	0.0089 (5)	0.0157 (6)
C15A	0.0524 (7)	0.0406 (6)	0.0545 (7)	0.0069 (5)	0.0036 (5)	0.0159 (5)
C16A	0.0701 (9)	0.0513 (8)	0.0729 (9)	0.0132 (7)	0.0212 (7)	0.0253 (7)
C17A	0.0810 (10)	0.0564 (8)	0.0768 (10)	0.0030 (7)	0.0147 (8)	0.0338 (8)
C18A	0.0732 (9)	0.0438 (7)	0.0702 (9)	0.0051 (6)	−0.0102 (7)	0.0244 (7)
C19A	0.1032 (14)	0.0499 (9)	0.1084 (15)	0.0054 (9)	−0.0141 (11)	0.0383 (10)
C20A	0.0604 (8)	0.0468 (7)	0.0932 (12)	0.0154 (6)	0.0052 (8)	0.0225 (8)
C21A	0.0531 (7)	0.0470 (7)	0.0823 (10)	0.0083 (6)	0.0102 (7)	0.0247 (7)
O1B	0.0694 (7)	0.0830 (8)	0.1104 (10)	−0.0131 (6)	−0.0198 (7)	0.0531 (8)
N1B	0.0501 (6)	0.0602 (7)	0.0635 (7)	0.0104 (5)	0.0081 (5)	0.0186 (6)
C1B	0.0523 (7)	0.0540 (8)	0.0599 (8)	0.0042 (6)	0.0050 (6)	0.0168 (6)
C2B	0.0788 (12)	0.0902 (13)	0.0716 (11)	0.0114 (10)	0.0026 (9)	−0.0038 (10)
C3B	0.0509 (7)	0.0525 (7)	0.0625 (8)	0.0060 (6)	0.0049 (6)	0.0270 (6)
C4B	0.0611 (8)	0.0504 (7)	0.0640 (8)	0.0143 (6)	0.0143 (6)	0.0285 (6)
C5B	0.0856 (12)	0.0815 (12)	0.0867 (12)	0.0396 (10)	0.0285 (10)	0.0370 (10)
C6B	0.0696 (8)	0.0479 (7)	0.0537 (7)	0.0072 (6)	0.0091 (6)	0.0247 (6)
C7B	0.1086 (14)	0.0711 (11)	0.0556 (9)	0.0200 (10)	0.0122 (9)	0.0214 (8)
C8B	0.1334 (19)	0.0982 (15)	0.0518 (9)	0.0051 (14)	−0.0100 (11)	0.0214 (10)
C9B	0.0911 (13)	0.1104 (16)	0.0705 (11)	−0.0019 (12)	−0.0183 (10)	0.0430 (11)
C10B	0.0625 (9)	0.0837 (11)	0.0719 (10)	0.0036 (8)	−0.0019 (7)	0.0385 (9)
C11B	0.0541 (7)	0.0531 (7)	0.0574 (8)	0.0019 (6)	0.0044 (6)	0.0260 (6)
C12B	0.0516 (7)	0.0651 (9)	0.0732 (9)	0.0038 (7)	0.0019 (7)	0.0323 (8)
C13B	0.0495 (7)	0.0686 (9)	0.0739 (10)	0.0077 (6)	−0.0026 (6)	0.0335 (8)
C14B	0.0484 (7)	0.0607 (8)	0.0552 (7)	0.0123 (6)	0.0045 (6)	0.0223 (6)
C15B	0.0509 (7)	0.0551 (7)	0.0480 (7)	0.0111 (6)	0.0086 (5)	0.0169 (6)
C16B	0.0554 (7)	0.0532 (7)	0.0601 (8)	0.0068 (6)	0.0009 (6)	0.0188 (6)
C17B	0.0604 (8)	0.0585 (8)	0.0576 (8)	0.0136 (6)	−0.0001 (6)	0.0197 (7)
C18B	0.0666 (8)	0.0575 (8)	0.0552 (8)	0.0148 (7)	0.0117 (6)	0.0213 (7)
C19B	0.0890 (12)	0.0681 (10)	0.0785 (11)	0.0127 (9)	0.0103 (9)	0.0357 (9)
C20B	0.0628 (8)	0.0626 (9)	0.0718 (10)	−0.0020 (7)	0.0057 (7)	0.0276 (8)
C21B	0.0516 (7)	0.0703 (9)	0.0621 (8)	0.0043 (6)	0.0021 (6)	0.0271 (7)

### Geometric parameters (Å, º)

**Table:**

O1A—C12A	1.2204 (18)	O1B—C12B	1.225 (2)
N1A—C1A	1.3132 (19)	N1B—C1B	1.3118 (19)
N1A—C11A	1.3621 (19)	N1B—C11B	1.3653 (19)
C1A—C3A	1.4248 (19)	C1B—C3B	1.421 (2)
C1A—C2A	1.497 (2)	C1B—C2B	1.504 (2)
C2A—H2AA	0.9600	C2B—H2BA	0.9600
C2A—H2AB	0.9600	C2B—H2BB	0.9600
C2A—H2AC	0.9600	C2B—H2BC	0.9600
C3A—C4A	1.371 (2)	C3B—C4B	1.373 (2)
C3A—C12A	1.5132 (19)	C3B—C12B	1.510 (2)
C4A—C6A	1.427 (2)	C4B—C6B	1.423 (2)
C4A—C5A	1.505 (2)	C4B—C5B	1.511 (2)
C5A—H5AA	0.9600	C5B—H5BA	0.9600
C5A—H5AB	0.9600	C5B—H5BB	0.9600
C5A—H5AC	0.9600	C5B—H5BC	0.9600
C6A—C11A	1.409 (2)	C6B—C11B	1.407 (2)
C6A—C7A	1.415 (2)	C6B—C7B	1.412 (2)
C7A—C8A	1.363 (3)	C7B—C8B	1.361 (3)
C7A—H7AA	0.9300	C7B—H7BA	0.9300
C8A—C9A	1.387 (3)	C8B—C9B	1.388 (3)
C8A—H8AA	0.9300	C8B—H8BA	0.9300
C9A—C10A	1.352 (3)	C9B—C10B	1.350 (3)
C9A—H9AA	0.9300	C9B—H9BA	0.9300
C10A—C11A	1.416 (2)	C10B—C11B	1.412 (2)
C10A—H10A	0.9300	C10B—H10B	0.9300
C12A—C13A	1.4586 (19)	C12B—C13B	1.463 (2)
C13A—C14A	1.330 (2)	C13B—C14B	1.334 (2)
C13A—H13A	0.9300	C13B—H13B	0.9300
C14A—C15A	1.4656 (18)	C14B—C15B	1.462 (2)
C14A—H14A	0.9300	C14B—H14B	0.9300
C15A—C21A	1.3897 (19)	C15B—C21B	1.391 (2)
C15A—C16A	1.391 (2)	C15B—C16B	1.397 (2)
C16A—C17A	1.383 (2)	C16B—C17B	1.379 (2)
C16A—H16A	0.9300	C16B—H16B	0.9300
C17A—C18A	1.380 (2)	C17B—C18B	1.388 (2)
C17A—H17A	0.9300	C17B—H17B	0.9300
C18A—C20A	1.381 (2)	C18B—C20B	1.385 (2)
C18A—C19A	1.511 (2)	C18B—C19B	1.502 (2)
C19A—H19A	0.9600	C19B—H19D	0.9600
C19A—H19B	0.9600	C19B—H19E	0.9600
C19A—H19C	0.9600	C19B—H19F	0.9600
C19A—H191	0.9601	C19B—H194	0.9600
C19A—H192	0.9600	C19B—H195	0.9599
C19A—H193	0.9599	C19B—H196	0.9600
C20A—C21A	1.381 (2)	C20B—C21B	1.382 (2)
C20A—H20A	0.9300	C20B—H20B	0.9300
C21A—H21A	0.9300	C21B—H21B	0.9300
			
C1A—N1A—C11A	118.55 (12)	C1B—N1B—C11B	118.75 (12)
N1A—C1A—C3A	122.70 (13)	N1B—C1B—C3B	122.47 (13)
N1A—C1A—C2A	117.16 (14)	N1B—C1B—C2B	116.23 (14)
C3A—C1A—C2A	120.13 (14)	C3B—C1B—C2B	121.30 (14)
C1A—C2A—H2AA	109.5	C1B—C2B—H2BA	109.5
C1A—C2A—H2AB	109.5	C1B—C2B—H2BB	109.5
H2AA—C2A—H2AB	109.5	H2BA—C2B—H2BB	109.5
C1A—C2A—H2AC	109.5	C1B—C2B—H2BC	109.5
H2AA—C2A—H2AC	109.5	H2BA—C2B—H2BC	109.5
H2AB—C2A—H2AC	109.5	H2BB—C2B—H2BC	109.5
C4A—C3A—C1A	119.84 (12)	C4B—C3B—C1B	119.88 (13)
C4A—C3A—C12A	120.88 (12)	C4B—C3B—C12B	121.83 (13)
C1A—C3A—C12A	118.97 (13)	C1B—C3B—C12B	118.24 (13)
C3A—C4A—C6A	118.14 (12)	C3B—C4B—C6B	118.39 (13)
C3A—C4A—C5A	121.59 (14)	C3B—C4B—C5B	121.84 (15)
C6A—C4A—C5A	120.26 (14)	C6B—C4B—C5B	119.76 (14)
C4A—C5A—H5AA	109.5	C4B—C5B—H5BA	109.5
C4A—C5A—H5AB	109.5	C4B—C5B—H5BB	109.5
H5AA—C5A—H5AB	109.5	H5BA—C5B—H5BB	109.5
C4A—C5A—H5AC	109.5	C4B—C5B—H5BC	109.5
H5AA—C5A—H5AC	109.5	H5BA—C5B—H5BC	109.5
H5AB—C5A—H5AC	109.5	H5BB—C5B—H5BC	109.5
C11A—C6A—C7A	118.12 (14)	C11B—C6B—C7B	117.93 (15)
C11A—C6A—C4A	117.95 (13)	C11B—C6B—C4B	117.77 (13)
C7A—C6A—C4A	123.92 (14)	C7B—C6B—C4B	124.30 (15)
C8A—C7A—C6A	120.88 (17)	C8B—C7B—C6B	120.77 (19)
C8A—C7A—H7AA	119.6	C8B—C7B—H7BA	119.6
C6A—C7A—H7AA	119.6	C6B—C7B—H7BA	119.6
C7A—C8A—C9A	120.62 (18)	C7B—C8B—C9B	120.97 (18)
C7A—C8A—H8AA	119.7	C7B—C8B—H8BA	119.5
C9A—C8A—H8AA	119.7	C9B—C8B—H8BA	119.5
C10A—C9A—C8A	120.38 (16)	C10B—C9B—C8B	120.01 (18)
C10A—C9A—H9AA	119.8	C10B—C9B—H9BA	120.0
C8A—C9A—H9AA	119.8	C8B—C9B—H9BA	120.0
C9A—C10A—C11A	120.90 (16)	C9B—C10B—C11B	120.87 (18)
C9A—C10A—H10A	119.5	C9B—C10B—H10B	119.6
C11A—C10A—H10A	119.5	C11B—C10B—H10B	119.6
N1A—C11A—C6A	122.79 (13)	N1B—C11B—C6B	122.73 (13)
N1A—C11A—C10A	118.11 (13)	N1B—C11B—C10B	117.81 (14)
C6A—C11A—C10A	119.10 (14)	C6B—C11B—C10B	119.46 (14)
O1A—C12A—C13A	120.78 (14)	O1B—C12B—C13B	120.41 (14)
O1A—C12A—C3A	118.55 (12)	O1B—C12B—C3B	120.02 (14)
C13A—C12A—C3A	120.66 (12)	C13B—C12B—C3B	119.55 (13)
C14A—C13A—C12A	123.47 (13)	C14B—C13B—C12B	124.80 (14)
C14A—C13A—H13A	118.3	C14B—C13B—H13B	117.6
C12A—C13A—H13A	118.3	C12B—C13B—H13B	117.6
C13A—C14A—C15A	126.58 (12)	C13B—C14B—C15B	126.41 (13)
C13A—C14A—H14A	116.7	C13B—C14B—H14B	116.8
C15A—C14A—H14A	116.7	C15B—C14B—H14B	116.8
C21A—C15A—C16A	117.72 (13)	C21B—C15B—C16B	117.63 (14)
C21A—C15A—C14A	123.02 (12)	C21B—C15B—C14B	119.55 (13)
C16A—C15A—C14A	119.26 (12)	C16B—C15B—C14B	122.81 (13)
C17A—C16A—C15A	121.02 (14)	C17B—C16B—C15B	120.76 (14)
C17A—C16A—H16A	119.5	C17B—C16B—H16B	119.6
C15A—C16A—H16A	119.5	C15B—C16B—H16B	119.6
C18A—C17A—C16A	121.22 (15)	C16B—C17B—C18B	121.68 (14)
C18A—C17A—H17A	119.4	C16B—C17B—H17B	119.2
C16A—C17A—H17A	119.4	C18B—C17B—H17B	119.2
C17A—C18A—C20A	117.75 (13)	C20B—C18B—C17B	117.47 (14)
C17A—C18A—C19A	121.40 (16)	C20B—C18B—C19B	121.82 (15)
C20A—C18A—C19A	120.84 (16)	C17B—C18B—C19B	120.72 (15)
C18A—C19A—H19A	109.5	C18B—C19B—H19D	109.5
C18A—C19A—H19B	109.5	C18B—C19B—H19E	109.5
C18A—C19A—H19C	109.5	C18B—C19B—H19F	109.5
C18A—C19A—H191	109.5	C18B—C19B—H194	109.5
C18A—C19A—H192	109.5	C18B—C19B—H195	109.5
H191—C19A—H192	109.5	H194—C19B—H195	109.5
C18A—C19A—H193	109.5	C18B—C19B—H196	109.5
H191—C19A—H193	109.5	H194—C19B—H196	109.5
H192—C19A—H193	109.5	H195—C19B—H196	109.5
C21A—C20A—C18A	121.71 (14)	C21B—C20B—C18B	121.45 (15)
C21A—C20A—H20A	119.1	C21B—C20B—H20B	119.3
C18A—C20A—H20A	119.1	C18B—C20B—H20B	119.3
C20A—C21A—C15A	120.59 (14)	C20B—C21B—C15B	121.00 (14)
C20A—C21A—H21A	119.7	C20B—C21B—H21B	119.5
C15A—C21A—H21A	119.7	C15B—C21B—H21B	119.5
			
C11A—N1A—C1A—C3A	−1.0 (2)	C11B—N1B—C1B—C3B	0.0 (2)
C11A—N1A—C1A—C2A	177.76 (15)	C11B—N1B—C1B—C2B	−179.83 (15)
N1A—C1A—C3A—C4A	−0.7 (2)	N1B—C1B—C3B—C4B	0.7 (2)
C2A—C1A—C3A—C4A	−179.44 (15)	C2B—C1B—C3B—C4B	−179.50 (16)
N1A—C1A—C3A—C12A	172.93 (13)	N1B—C1B—C3B—C12B	178.29 (13)
C2A—C1A—C3A—C12A	−5.8 (2)	C2B—C1B—C3B—C12B	−1.9 (2)
C1A—C3A—C4A—C6A	1.7 (2)	C1B—C3B—C4B—C6B	−0.8 (2)
C12A—C3A—C4A—C6A	−171.85 (12)	C12B—C3B—C4B—C6B	−178.34 (13)
C1A—C3A—C4A—C5A	−179.44 (15)	C1B—C3B—C4B—C5B	177.93 (14)
C12A—C3A—C4A—C5A	7.0 (2)	C12B—C3B—C4B—C5B	0.4 (2)
C3A—C4A—C6A—C11A	−1.0 (2)	C3B—C4B—C6B—C11B	0.33 (19)
C5A—C4A—C6A—C11A	−179.88 (15)	C5B—C4B—C6B—C11B	−178.44 (13)
C3A—C4A—C6A—C7A	178.27 (15)	C3B—C4B—C6B—C7B	−179.03 (14)
C5A—C4A—C6A—C7A	−0.6 (2)	C5B—C4B—C6B—C7B	2.2 (2)
C11A—C6A—C7A—C8A	0.2 (3)	C11B—C6B—C7B—C8B	0.1 (3)
C4A—C6A—C7A—C8A	−179.05 (18)	C4B—C6B—C7B—C8B	179.43 (18)
C6A—C7A—C8A—C9A	0.6 (4)	C6B—C7B—C8B—C9B	0.5 (3)
C7A—C8A—C9A—C10A	−1.1 (4)	C7B—C8B—C9B—C10B	−0.5 (4)
C8A—C9A—C10A—C11A	0.8 (3)	C8B—C9B—C10B—C11B	0.0 (3)
C1A—N1A—C11A—C6A	1.7 (2)	C1B—N1B—C11B—C6B	−0.5 (2)
C1A—N1A—C11A—C10A	−177.83 (14)	C1B—N1B—C11B—C10B	179.82 (14)
C7A—C6A—C11A—N1A	179.97 (15)	C7B—C6B—C11B—N1B	179.75 (14)
C4A—C6A—C11A—N1A	−0.7 (2)	C4B—C6B—C11B—N1B	0.4 (2)
C7A—C6A—C11A—C10A	−0.5 (2)	C7B—C6B—C11B—C10B	−0.6 (2)
C4A—C6A—C11A—C10A	178.82 (13)	C4B—C6B—C11B—C10B	−179.99 (13)
C9A—C10A—C11A—N1A	179.55 (17)	C9B—C10B—C11B—N1B	−179.75 (16)
C9A—C10A—C11A—C6A	0.0 (3)	C9B—C10B—C11B—C6B	0.6 (2)
C4A—C3A—C12A—O1A	93.91 (19)	C4B—C3B—C12B—O1B	106.73 (19)
C1A—C3A—C12A—O1A	−79.67 (19)	C1B—C3B—C12B—O1B	−70.8 (2)
C4A—C3A—C12A—C13A	−85.26 (18)	C4B—C3B—C12B—C13B	−74.90 (19)
C1A—C3A—C12A—C13A	101.16 (17)	C1B—C3B—C12B—C13B	107.53 (17)
O1A—C12A—C13A—C14A	172.52 (17)	O1B—C12B—C13B—C14B	168.76 (17)
C3A—C12A—C13A—C14A	−8.3 (3)	C3B—C12B—C13B—C14B	−9.6 (3)
C12A—C13A—C14A—C15A	178.97 (14)	C12B—C13B—C14B—C15B	−179.21 (14)
C13A—C14A—C15A—C21A	−7.3 (2)	C13B—C14B—C15B—C21B	−177.55 (15)
C13A—C14A—C15A—C16A	173.14 (16)	C13B—C14B—C15B—C16B	1.9 (2)
C21A—C15A—C16A—C17A	0.2 (2)	C21B—C15B—C16B—C17B	0.4 (2)
C14A—C15A—C16A—C17A	179.76 (15)	C14B—C15B—C16B—C17B	−179.05 (13)
C15A—C16A—C17A—C18A	0.4 (3)	C15B—C16B—C17B—C18B	0.4 (2)
C16A—C17A—C18A—C20A	−0.6 (3)	C16B—C17B—C18B—C20B	−0.4 (2)
C16A—C17A—C18A—C19A	178.33 (17)	C16B—C17B—C18B—C19B	179.86 (14)
C17A—C18A—C20A—C21A	0.2 (3)	C17B—C18B—C20B—C21B	−0.3 (2)
C19A—C18A—C20A—C21A	−178.73 (16)	C19B—C18B—C20B—C21B	179.41 (15)
C18A—C20A—C21A—C15A	0.4 (3)	C18B—C20B—C21B—C15B	1.1 (2)
C16A—C15A—C21A—C20A	−0.6 (2)	C16B—C15B—C21B—C20B	−1.1 (2)
C14A—C15A—C21A—C20A	179.86 (15)	C14B—C15B—C21B—C20B	178.35 (14)

### Hydrogen-bond geometry (Å, º)

**Table:**

*D*—H···*A*	*D*—H	H···*A*	*D*···*A*	*D*—H···*A*
C16*B*—H16*B*···O1*A*^i^	0.93	2.63	3.4465 (19)	148

Symmetry code: (i) *x*+1, *y*, *z*.

## References

[bb1] Agilent (2012). *CrysAlis PRO* Agilent Technologies, Yarnton, England.

[bb2] Campbell, S. F., Hardstone, J. D. & Palmer, M. J. (1998). *J. Med. Chem.* **31**, 1031–1035. 10.1021/jm00400a025 2896245

[bb3] Dimmock, J. R., Elias, D. W., Beazely, M. A. & Kandepu, N. M. (1999). *Curr. Med. Chem.* pp. 1125–1149. 10519918

[bb4] Kalluraya, B. & Sreenivasa, S. (1998). *Farmaco*, **53**, 399–404. 10.1016/s0014-827x(98)00037-8 9764472

[bb5] Muscia, G. C., Bollini, M., Carnevale, J. P., Bruno, A. M. & Asis, S. E. (2006). *Tetrahedron Lett.* **47**, 8811–8815.

[bb6] Prasath, R., Bhavana, P., Butcher, R. J. & Jasinski, J. P. (2011). *Acta Cryst.* E**67**, o621. 10.1107/S1600536811004661 PMC3051952 21522377

[bb7] Sheldrick, G. M. (2008). *Acta Cryst.* A**64**, 112–122. 10.1107/S0108767307043930 18156677

[bb8] Xiang, W., Tiekink, E. R. T., Iouri, K., Nikolai, K. & Mei, L. G. (2006). *Eur. J. Pharm. Sci.* **27**, 175–187.

